# Evaluation of the impacts of photodynamic therapy on the prognosis of patients with hrHPV infection based on BTNL8 expression

**DOI:** 10.3389/fonc.2023.1218808

**Published:** 2023-06-29

**Authors:** Hongqing Lv, Shuai Lou, Lin Zhang, Dawei Cui, Yao Li, Ying Yang, Meilan Chen, Pan Chen

**Affiliations:** ^1^Department of Gynecology, Jinhua Municipal Central Hospital, Jinhua, Zhejiang, China; ^2^Department of Gynecology, Jinhua Maternal and Child Health Hospital, Jinhua, Zhejiang, China

**Keywords:** high-risk HPV infection, photodynamic therapy, BTNL8, prognosis evaluation, HPV

## Abstract

**Objective:**

The aim of this study was to evaluate the prognostic value of Butyrophilin-like protein 8 (BTNL8) expression in high-risk HPV (hrHPV) infection treated with photodynamic therapy.

**Methods:**

A total of 93 patients with hrHPV infection were enrolled as research study subjects, along with 69 healthy women who served as controls. Serum samples were obtained from each participant, and BTNL8 levels were quantified. The patients were divided into high- and low-expression groups (*n* = 45 and *n* = 48, respectively), and both groups underwent photodynamic therapy. We recorded the following data: BTNL8 expression pre-treatment and at 3/6 months post-treatment, HPV negative conversion ratio, regression rate of low-grade squamous intraepithelial lesions (LSIL), incidence of adverse reactions, complication rate, serum inflammatory factors, persistence of HPV positivity, LSIL residue or recurrence, and incidence of high-grade cervical intraepithelial lesions (HCIL).

**Results:**

Patients with HPV infection exhibited higher BTNL8 expression than healthy individuals. Compared to the low-expression group, the high-expression group showed increased HPV negative conversion ratios, LSIL regression rates, and levels of IL-17 and IL-23. This group also demonstrated decreased total complication rate, HPV positivity persistence, LSIL residue or recurrence, and IL-10 levels. Additionally, there was no significant difference between the two groups in terms of the number of adverse reactions and cases with LSIL residue/recurrence.

**Conclusion:**

Serum BTNL8 expression may serve as a valuable tool for early screening and prognosis monitoring of patients with hrHPV infection.

## 1 Introduction

Cervical cancer (CC) is the fourth most commonly diagnosed cancer in women. In 2020, approximately 604,127 new cases were reported globally, accounting for 3.1% of all cancer diagnoses, making it the ninth most common type of cancer (1). China, in particular, faces a significant challenge due to its high incidence rate. As per recent statistics, 106,000 confirmed cases of CC and 48,000 related deaths were reported in China in 2018 (2). Notably, high-risk human papillomavirus (hrHPV) infection is associated with up to 90% of all CC cases.

HPV, a small double-stranded DNA virus, circumvents the host’s innate immunity and integrates its genome into the host cell (3). When hrHPV infection persists, it causes its structural oncoproteins E6 and E7 to contribute to the inactivation of the tumor suppressor proteins p53 and pRB, thereby disrupting the normal functioning of DNA repair and apoptosis and triggering abnormal proliferation of normal cervical epithelial squamous cells, known as cervical intraepithelial neoplasia (CIN) (4–6). If left untreated, unresolved CIN1 will continue to progress into high-grade squamous intraepithelial lesions (HSIL), with massive genomic alterations that induce CC occurrence (7).

The treatment options for hrHPV infection include circumcision, cryotherapy, conization, therapeutic vaccine, and photodynamic therapy (8). Traditional treatment methods, which mainly encompass circumcision, cryotherapy, and conization, present some shortcomings, such as high recurrence rate, serious tissue damage, and potential structural destruction of cervical if improperly applied (9). Photodynamic therapy, a novel cancer treatment modality, involves the activation of photosensitive molecules by lasers of a specific wavelength. The activated photosensitive molecules prompt the production of reactive oxygen species (ROS) leading to irreversible cell apoptosis and necrosis, and consequently inhibiting the proliferation and invasion of cancer cells (10, 11). Photodynamic therapy offers advantages in fostering good tissue healing and the convenient utilization of light (12). Cang et al. (13) pointed out the potential of photodynamic therapy to effectively eliminate hrHPV infection, particularly HPV16 and HPV18. Gu et al. (14) propose that 5-aminolevulinic acid-mediated photodynamic therapy, an effective treatment for hrHPV infection co-occurring with cervical squamous intraepithelial lesions, is less likely to cause cervical injury. Therefore, photodynamic therapy emerges as a promising, effective therapy for hrHPV infection.

During the course of hrHPV infection, genes such as p16 and galectin-3 may demonstrate aberrant expression, which could serve as biomarkers of HPV infection for early screening for cervical lesions (15, 16). Thus, incorporating gene expression into the prognostic evaluation of hrHPV infections can facilitate dynamic detection post-therapy and expedite the investigation and treatment of recurrent cases. Butyrophilin-like protein 8 (BTNL8) is implicated in adaptive immune process and thus serves as a crucial regulatory factor of tumor immunity (17–19). Yang et al. (20) observed a significant correlation of BTNL8 expression with patients’ survival rate according to the relatively low survival rate in CC patients with high BTNL8 expression (<40%) and the over 60% of survival rate in CC patients with low expression. The results suggest BTNL8 as one possible biomarker for the prognosis of CC and its potential for postoperative evaluation of hrHPV infection.

As of now, the potential evaluative significance of BTNL8 in HPV infection has not been reported. Therefore, this study aims to assess the clinical efficacy and postoperative recurrence of photodynamic therapy in hrHPV infection through the lens of BTNL8, hoping to provide a reliable basis for the clinical dynamic monitoring of hrHPV infection.

## 2 Methods

### 2.1 General data

A total of 93 women diagnosed with hrHPV infection at Jinhua Central Hospital from January 2020 to March 2022 were included in this study. The inclusion criteria were as follows: patients confirmed with hrHPV infection according to the PCR method (21) and cervical cytology (22), patients with detailed clinical data, patients not in pregnancy and lactation period, and those who were willing to participate in the research. The exclusion criteria were as follows: patients who had received HPV infection therapy; patients who had received immunosuppressive agents or glucocorticoids recently; patients with comorbid CC or other malignant tumors of the reproductive system; patients with comorbid dysfunction of the heart, lung, liver, or kidney; patients with comorbid mental disorder; and those allergic to photodynamic therapy. A total of 69 healthy women were enrolled as controls. All participants of the study offered signed informed consent forms after understanding the whole process of the study, and approval was obtained for the study from the ethics committee of our hospital.

### 2.2 Photodynamic therapy

5-Aminolevulinic acid-mediated photodynamic therapy was adopted for treating patients with hrHPV infection in this study. Specifically, each patient was given photodynamic therapy at 7 days after the menstrual period. Before therapy, the patient was required to empty his/her bladder to avoid interference. Start with a surface shave, the lesions were cleaned *via* normal saline and then given ultra-expensive disinfection, followed by even application of 20% 5-aminolevulinic acid (Shanghai Fudan-Zhangjiang Bio-Pharmaceutical Co., Ltd, 118 mg/piece) with cotton swabs (23). After 3 h, the lesions were irradiated with a Y-HN300 He-Ne laser therapy machine (Wuhan Yage Laser Equipment Co., Ltd.) (output wavelength: 632.8 nm; rated power: 300 mW) at 20 min each time, once every 7 days, for four consecutive treatments, and the observation course was 6 months.

### 2.3 Outcome measures

(1) Clinical efficacy: Patients are asked to visit the hospital for regular follow-up at 3 months and 6 months after surgery. The negative convention of HPV and inversion of low-grade squamous intraepithelial lesions (LSIL) in patients at 3 months and 6 months after surgery were recorded. The LSIL residue/recurrence of LSIL lesions (the cervicovaginal part and the cervicovaginal part + cervical canal) at 6 months after surgery was evaluated and recorded.
(2) Serum BTNL8 expression: Serum was sampled from each patient with hrHPV infection before admission, and serum was also acquired from healthy women as controls. Serum was sampled from every patient before therapy and at 3 and 6 months after therapy. All blood specimens were acquired on an empty stomach. Total RNA of sampled serum was acquired *via* a plasma/serum RNA extraction kit (NORGEN, Canada), followed by fluorescent quantitation *via* a qPCR instrument. The design and synthesis of BTNL8 primers were completed by Shanghai Sangon Biotech Co., Ltd. The 2^−ΔΔCt^ method was adopted for standardizing the gene expression.
(3) Serum inflammatory factor levels: Serum inflammatory factors were quantified *via* ELISA kits of IL-17 (ab100556), IL-23 (ab221837), and IL-10 (ab185986) (Abcam, Shanghai) at 3 months after surgery.
(4) Adverse reactions and complications: The adverse reactions and complications of patients within 3 months after surgery were recorded. Adverse reactions included colporrhagia, menstrual abdominal pain, and colpitis. Postoperative complications included bleeding, infection, menstrual disorder, prolonged menstrual period, and cervical duct adhesion.
(5) Prognostic indexes: Prognostic indexes at 6 months after surgery were recorded, including persistence in positive HPV, LSIL residue/recurrence, and progression to high-grade cervical intraepithelial lesions (HCIL).

### 2.4 Statistical analyses

This study adopted SPSS 20.0 for statistical analyses of data. Counting data were presented by *n* (%), and measurement data were presented by mean ± SD, all of which were analyzed *via* the normality test. The independent-samples *t*-test was utilized for inter-group comparison and the chi-square test was used for inter-group comparison of counting data, and the repeated measures analysis of variance was used for intra-group comparison. 95% CI was adopted. *p* < 0.05 implies a notable difference.

## 3 Results

### 3.1 Aberrant serum BTNL8 expression in cases with hrHPV infection

**Figure 1:**
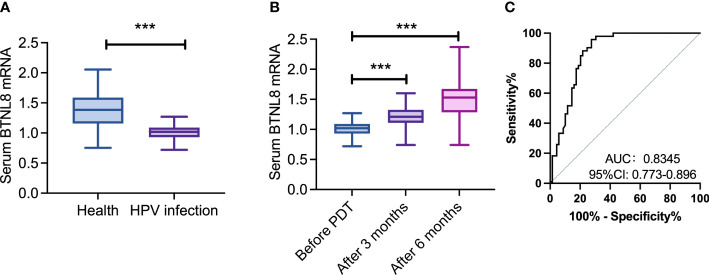
Aberrant serum BTNL8 expression in cases with hrHPV infection. **(A)** Comparison of serum BTNL8 expression levels between patients and healthy women. **(B)** Comparison of serum BTNL8 expression between patients after photodynamic therapy 3 months and before. **(C)** Comparison of serum BTNL8 expression levels between patients before and after photodynamic therapy for 6 months. *** vs. *p* < 0.001.

A total of 93 patients with hrHPV infection and 69 healthy women were enrolled, and compared in serum BTNL8 expression (**Figure 1**). The patients showed notably downregulated serum BTNL8 than the healthy women (*p* < 0.05). In contract to serum BTNL8 expression before therapy, the HPV infection group showed notably up-regulated BTNL8 expression at 3 and 6 months after photodynamic therapy (*p* < 0.05). ROC curves revealed the potential value of serum BTNL8 in screening hrHPV infection (AUC = 0.835, *p* < 0.05).

### 3.2 Correlation of serum BTNL8 expression with clinical efficacy of photodynamic therapy for HPV infection

Patients were assigned to the high- and low-expression groups (*n* = 45 and 48, respectively) based on the median relative expression level of serum BTNL8 obtained prior to admission (1.02). The relative expression levels of BTNL8 in the two groups were 1.11 ± 0.06 and 0.93 ± 0.08, respectively. The former group consisted of patients at 35.69 ± 6.73 years old, with a course of disease of 15.76 ± 5.20 months and body mass index (BMI) of 20.72 ± 1.76 kg/m^2^, including 33 cases of cervicovaginal attacks and 12 cases of attacks in the cervicovaginal part + cervical canal. The latter group consisted of patients at 36.24 ± 6.20 years old, with a course of disease of 13.84 ± 4.54 months and a body mass index (BMI) of 20.99 ± 1.70 kg/m^2^, including 37 cases of cervicovaginal attacks and 11 cases of attacks in the cervicovaginal part + cervical canal. The two groups were similar in general data (**Table 1**).

**Table 1:** General data.

	High-expression group (*n* = 45)	Low-expression group (*n* = 48)	*χ*^2^/t	*p*
Age (years)	35.69 ± 6.73	36.24 ± 6.20	0.421	0.675
Course of disease (months)	15.76 ± 5.20	13.84 ± 4.54	1.851	0.067
Body mass index (kg m^−2^	20.72 ± 1.76	20.99 ± 1.70	0.764	0.447
Lesion site			0.176	0.675
Cervicovaginal part	33 (73.33)	37 (77.08)		
Cervicovaginal part + cervical canal	12 (26.67)	11 (22.92)		

**Figure 2:**
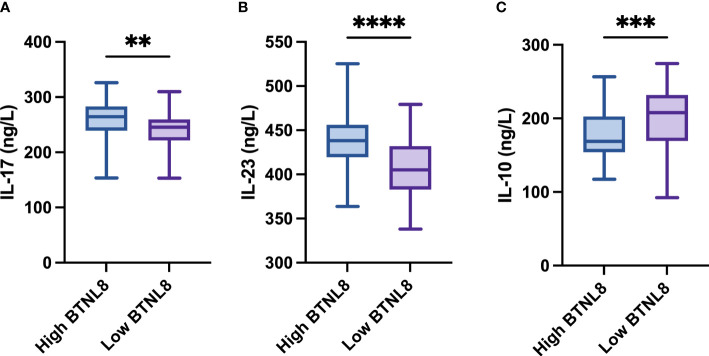
Comparison of BTNL8 expression with postoperative serum inflammatory factors. Comparison of BTNL8 expression with postoperative serum **(A)** IL-17 levels; **(B)** IL-23; **(C)** IL-10 levels. ** vs. *p* < 0.01; *** vs. *p* < 0.001; **** vs. *p* < 0.0001.

The clinical efficacy at 3 and 6 months after photodynamic therapy were counted (**Tables 2**, **3**). At 3 and 6 months after therapy, the high-expression group presented notably higher negative conversion ratio of HPV and inversion rate of LISL than the other (both *p* < 0.05). The quantification results of serum inflammatory factors in postoperative patients revealed notably higher IL-17 and IL-23 levels and a notably lower IL-10 level in the high-expression group than in the other group (all *p* < 0.04, **Figure 2**).

**Table 2:** Association of BTNL8 expression with negative conversion ratio of HPV.

	At 3 months after treatment	At 6 months after treatment
High-expression group (*n* = 45)	35 (77.78)	37 (82.22)
Low-expression group (*n* = 48)	28 (58.33)	30 (62.50)
*χ*^2^	4.019	4.485
*p*-value	0.045	0.034

**Table 3:** Association of BTNL8 expression with inversion of LSIL.

	At 3 months after treatment	At 6 months after treatment
High-expression group (*n* = 45)	34 (75.56)	36 (80.00)
Low-expression group (*n* = 48)	26 (54.17)	27 (56.25)
*χ*^2^	4.641	5.995
*p*-value	0.031	0.014

### 3.3 Associations of serum BTNL8 expression with postoperative adverse reactions and complications

**Figure 3:**
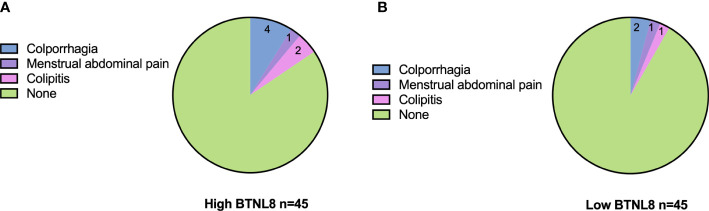
Associations of serum BTNL8 expression with postoperative adverse reactions. Distribution of postoperative adverse effects in patients in the **(A)** high BTNL8 expression group versus those in the **(B)** low BTNL8 expression group.

The postoperative adverse reactions (colporrhagia, menstrual abdominal pain, and colpitis) and complications in patients were counted. The low-expression group presented a total incidence of adverse reactions of 10.42%, with two cases of colporrhagia, two cases of menstrual abdominal pain, and one case of colpitis, while the other group presented a total incidence of adverse reactions of 9.76%, with two cases of colporrhagia, one case of menstrual abdominal pain, and one case of colpitis (**Figure 3**). The two groups were similar in the incidence of adverse reactions (*p* > 0.05).

The associations of serum BTNL8 with postoperative complications (bleeding, infection, menstrual disorder, prolonged menstrual period, and cervical duct adhesion) were analyzed (**Table 4**). The low-expression group presented a total incidence of complications of 29.17%, with one case of bleeding, two cases of infection, four cases of menstrual disorder, five cases of prolonged menstruation, and two cases of cervical duct adhesion, while the other group presented a total incidence of complications of 11.11%, with one case of bleeding, one case of infection, two cases of menstrual disorder, no case of prolonged menstruation, and one case of cervical duct adhesion. The latter group presented a notably lower incidence than the former group.

**Table 4:** Association of BTNL8 expression with postoperative complications.

	Bleeding	Infection	Menstrual disorder	Prolonged menstruation	Cervical duct adhesion	Total incidence of complications
High-expression group (*n* = 45)	1 (2.22)	1 (2.22)	2 (4.44)	0	1 (2.22)	5 (11.11)
Low-expression group (*n* = 48)	1 (2.08)	2 (4.17)	4 (8.33)	5 (10.42)	2 (4.17)	14 (29.17)
*χ*^2^						4.658
*p*-value						0.031

### 3.4 Association of serum BTNL8 with postoperative survival outcome

The associations of BTNL8 expression with persistence in positive HPV, LSIL residue/recurrence, and HCIL were analyzed, and the results revealed 4 cases of persistent positive HPV (8.89%), 6 cases of LSIL residue/recurrence (12.50%), and 0 cases of progression to HCIL in the high-expression group and 12 cases of persistent positive HPV (25.00%), 15 cases of LSIL residue/recurrence (31.25%), and 0 cases of progression to HCIL in the other group (**Table 5**). The two groups were notably different in persistence in positive HPV and LSIL residue/recurrence (both *p* < 0.05).

**Table 5:** Association of serum BTNL8 with postoperative survival outcome.

	Persistent HPV positive	LSIL residue/recurrence	Progression to HCIL
High-expression group (*n* = 45)	4(8.89)	6 (12.50)	0
Low-expression group (*n* = 48)	12 (25.00)	15 (31.25)	0
*χ*^2^	4.232	4.265	–
*p*-value	0.040	0.039	–

The associations of BTNL8 expression with residual/recurrent sites of LSIL were also analyzed, and the results revealed two cases in the cervicovaginal part (4.44%) and four cases in the cervical part (8.89%) in the high-expression group, and six cases in the cervicovaginal part (12.50%) and nine cases in the cervicovaginal part + cervical canal (18.75%) in the other group (**Table 6**). The two groups were similar in the incidence rate in the cervicovaginal part and cervicovaginal part + cervical canal (*p* > 0.05).

**Table 6:** Association of serum BTNL8 with LSIL residue/recurrence.

	Cervicovaginal part	Cervicovaginal part + cervical canal
High-expression group (*n* = 45)	2(4.44)	4 (8.89)
Low-expression group (*n* = 48)	6 (12.50)	9 (18.75)
*χ*^2^	1.917	1.878
*p*	0.166	0.171

## 4 Discussion

Screening for hrHPV infection is a crucial link to prevent CC. At the current stage, hrHPV infection is mainly detected *via* membrane liquid-based ultrathin cytology, HPV-DNA detection, and colposcopy biopsy. With features of convenient and simple operation, serum marker-based detection can be adopted as one auxiliary means of the above methods, which is helpful for postoperative dynamic monitoring of patients. Troja et al. (24) believe a strong association of serum vitamin D with hrHPV infection. BTNL8 has been verified to be bound up with the survival of CC (20). Thus, we adopted it for evaluating clinical significance of photodynamic therapy for hrHPV infection. Our results imply the positive role of serum BTNL8 for the evaluation.

Firstly, we found downregulated BTNL8 in serum samples of patients with hrHPV infection and the induction of photodynamic therapy to BTNL8 expression, which may be attributed to the regulatory influence of BTNL8 on the immune system. In principle, photodynamic therapy triggers DNA damage, apoptosis, and necrosis through the production of ROS, during which it also stimulates a series of immune responses to clear cells (25, 26). BTNL8 happens to be one crucial factor associated with immune system, which mediates the dependent selection of T-cell receptors such as Vγ4+/Vδ1+ intraepithelial lymphocytes and γδT cells (27, 28). Thus, under the influence of photodynamic therapy on immune response in the tumor microenvironment, BTNL8 expression changes and acts as a key factor to regulate the proliferation of associated immune cells, which is probably the reason for the increase of BTNL8 under photodynamic forces. Furthermore, the high efficacy accompanied by high BTNL8 expression and the association of the high expression with serum inflammatory factors also verify the possible involvement of BTNL8 in the immune mechanism under photodynamic therapy for hrHPV infection. The specific function of BTNL8 will be discussed in future research. According to ROC-based analysis, BTNL8 facilitates distinguishing hrHPV infection. The results imply that serum BTNL8 is promising in the triage and management of HPV-positive patients to lower unnecessary colposcopy referrals and other related injuries (29).

In our investigation, we did not discern a significant correlation between BTNL8 expression and the occurrence of postoperative adverse events. However, the limited sample size of our study might obscure more nuanced impacts, potentially introducing selection bias and limiting the validity of these findings. In future investigations, we plan to enlarge our sample size, which will enable a more comprehensive and reliable analysis of the potential association between BTNL8 expression and postoperative adverse reactions in the context of hrHPV infection. Interestingly, our data pointed towards an inverse relationship between BTNL8 expression and complication incidence. Recurrence symptoms following photodynamic therapy in hrHPV-infected patients can manifest as persistent HPV positivity, residual or recurrent LSIL, or progression to HCIL (30–33).

Within our study cohort, no patients advanced to HCIL post-photodynamic therapy. Nonetheless, a higher BTNL8 expression was associated with significantly reduced instances of LSIL recurrence and persistent HPV positivity. It should be noted that we have not accounted for potential confounding factors that could impact BTNL8 expression within this study. Furthermore, we did not identify a correlation between serum BTNL8 expression and the location of LSIL residue or recurrence. This suggests that while serum BTNL8 levels may serve as an indicator of residual or recurrent LSIL, it does not appear to facilitate identification of the specific site of these conditions. This aspect warrants further exploration in future research.

To sum up, this study suggests the associations of serum BTNL8 expression with the clinical efficacy, complications, persistent positive HPV, and LSIL residue/recurrence in the therapy of HPV infection by photodynamic therapy, which is helpful to dynamically monitor the prognosis of patients, timely screen patients with recurrence risk, and potentially facilitate biomarker-based triage and management of HPV-positive patients.

## Data availability statement

The original contributions presented in the study are included in the article/supplementary material. Further inquiries can be directed to the corresponding authors.

## Ethics statement

The studies involving human participants were reviewed and approved by Ethics Committee of Jinhua Central Hospital. The patients/participants provided their written informed consent to participate in this study.

## Author contributions

HL, SL, and LZ contributed to conception and design of the study. HL organized the data. DC performed the statistical analysis. YL wrote the first draft of the manuscript. YY, MC, and PC wrote sections of the manuscript. All authors contributed to the article and approved the submitted version.
